# The impact of smoking on estimated biological age and body fat composition: A cross-sectional study

**DOI:** 10.18332/tid/174663

**Published:** 2023-12-06

**Authors:** Goranka Radmilović, Valentina Matijević, Danijel Mikulić, Danijela Rašić Markota, Asija Rota Čeprnja

**Affiliations:** 1 County General Hospital Požega, Požega, Croatia; 2 Faculty of Medicine, Josip Juraj Strossmayer University of Osijek, Osijek, Croatia; 3 Clinic for Rheumatology, Physical Medicine and Rehabilitation, Clinical Hospital Centre Sestre Milosrdnice, Zagreb, Croatia; 4 Libertas International University, Zagreb, Croatia; 5 School of Medicine, Catholic University of Croatia, Zagreb, Croatia; 6 Unified Emergency Hospital Admission, Clinical Hospital Centre Sestre Milosrdnice, Zagreb, Croatia; 7 Health Centre Zagreb, Zagreb, Croatia; 8 Department of Physical Medicine and Rehabilitation, University Hospital of Split, Split, Croatia

**Keywords:** aging, body composition, healthy aging, tobacco smoking

## Abstract

**INTRODUCTION:**

Smoking tobacco results in the death of more than 8 million people every year. Our study aims to explore a new approach for smoking cessation by analyzing body composition differences between smokers and non-smokers, focusing on potential variations in biological and chronological ages.

**METHODS:**

A cross-sectional study was conducted in 2019 at the Special Hospital for Medical Rehabilitation in Croatia. It included 164 subjects, 81 smokers and 83 non-smokers, aged 40–65 years. This study was part of a two-year investigation on locomotor parameter changes as early COPD predictors. Measurements encompassed body parameters, spirometry tests, and body composition. Spirometry was done using the Flowscreen Pro device, and the FEV1/FVC ratio determined lung function. The GAIA 359 PLUS device assessed body composition and estimated biological age. Exclusion criteria applied to specific medical conditions and recent surgeries.

**RESULTS:**

Smokers had a slightly higher percentage of fat tissue than non-smokers (median=27%, IQR: 24–31) compared to non-smokers (median=25%, IQR: 22–28, p<0.001). The difference in the Gaia estimated age and the actual age of the subjects was significantly higher in the group of smokers (median=2, IQR: 0–3) compared to non-smokers (median=0 IQR: -2–2, p=0.003), but they did not differ in muscle tissue, weight or the proportion of trunk adipose tissue.

**CONCLUSIONS:**

Our study revealed that smokers exhibit higher fat tissue percentages and a higher estimated biological age relative to non-smokers. These findings underscore the established health hazards of smoking and the imperative for smoking cessation in mitigating these adverse effects.

## INTRODUCTION

Over years of extensive global data accumulation, the critical nexus between tobacco product usage and health ramifications has emerged, catalyzed by pioneering observations like the Doll and Hill^1^ link between tobacco and lung cancer. Medical research serves dual objectives – treating ailments and preemptively averting them – underscoring the ascendancy of proactive awareness and prevention over belated corrective measures. Acknowledging tobacco’s pivotal role as a risk factor in lethal disease, a concerted drive to reduce smoking prevalence and consumption has become paramount.

Epidemiological evidence underscores the dire impact of the cigarette smoking epidemic, causing 8 million annual deaths worldwide, with 1.2 million attributed to passive smoking^2^. Stringent regulations have curbed passive smoking, exemplified by the reduction in workplace exposure from 31.9% to 2.5% by 2014^3^. While over 80% of smokers live in developing countries, the global prevalence amounts to 1.7 billion individuals (22.3% of the global population), with varying gender ratios^2,4^.

In the realm of body composition, age-related changes yield pronounced shifts: the aging process amplifies fat tissue percentages while depleting muscle mass and bone density, notwithstanding steady substance proportions^5^. Cigarette smoke’s deleterious effects span over 4000 chemicals, provoking an inflammatory response and impairing ATP synthesis, leading to muscular atrophy and diminished skeletal muscle endurance^6,7^. Smoking’s association with osteoporosis is evident through disruption of bone remodeling mechanisms via the RANKL-RANK-OPG pathway^8^. The enigma of lower body mass and body mass index (BMI, kg/m^2^) among smokers can be attributed to nicotine’s multifaceted impact, including appetite reduction and altered metabolism, albeit not indicative of a healthful diet^9-11^. Smoking increases the waist-to-hip ratio (WHR), an indicator of visceral adiposity linked to metabolic syndrome, diabetes, and cardiovascular disease^12^.

Advancing age stands as a hallmark risk factor for chronic diseases, yet it does not universally predict individual health outcomes, accentuating age-related heterogeneity^13^. As the world population ages, understanding biological aging gains urgency, with the concept of biological age captivating public interest. The GAIA 359 PLUS emerges as a tool to gauge biological age through body composition assessment.

In this context, our research aims to investigate a novel approach to facilitate smoking cessation among healthy individuals by analyzing body mass composition differences between smokers and non-smokers, with a focus on discerning potential variations in their biological and chronological ages.

## METHODS

A cross-sectional study was conducted on patients undergoing medical rehabilitation or medically programmed vacation at the Special Hospital for Medical Rehabilitation in Daruvarske Toplice, Croatia in 2019. The patients were part of another, large two-year study that investigated changes in locomotor parameters in smokers as an early predictor for the development of COPD. The sample included a total of 164 subjects, 81 smokers and 83 non-smokers. The age of the subjects was between 40 and 65 years, and all patients voluntarily signed an informed consent to participate in the study. The Ethical Committee of the Special Hospital for Medical Rehabilitation Daruvarske Toplice gave consent to conduct a larger study registered at Clinicaltrials.gov under the study’s ID NCT04643600, of which this study is a part. Body mass, height, BMI and waist circumference were measured for all patients. All patients underwent spirometry, and patients whose Tiffeneau–Pinelli index (the ratio of FEV1 to FVC) was lower than 70%, i.e. those with a diagnosis of COPD, were excluded from the study. Excluded from the sample were patients suffering from inflammatory rheumatic diseases, malignant diseases, acute and severe heart or lung diseases, unregulated hypertension, those who underwent a major surgical procedure in the past year, as well as patients with implanted hip, knee or ankle prostheses.

All subjects were spirometrically tested on the device Flowscreen Pro from the brand Jaeger according to the guidelines of the European Respiratory Society^14^. Each subject underwent three spirometric tests, with prior education on how to perform that test, and the highest recorded value was taken as a parameter, which was expressed as a percentage of the expected value. To assess lung function, the FEV1/FVC ratio indicator was taken, and a value ≥0.70 was taken as a value that shows that the subject does not have developed airway obstruction^15^. Any subject who had a lower value on the spirometric test was excluded from the study.

Body composition measurements were obtained using the GAIA 359 PLUS device, which has been employed in several research studies due to its proven reproducibility and reliability in the assessment of body composition^16-18^. The device works on the principle of measuring the resistance of the human body when alternating current is passed through it. The measurement is carried out by the subject standing barefoot in the intended place and taking the sticks with both hands with the thumbs placed in the appropriate places, moving the hands away from the body at an angle of 45 degrees with the elbows outstretched, and remaining still in that position for ten seconds until the device finishes reading the value. Based on the composition of the body mass, the device also estimates the biological age of the subject in years.

Demographic inquiries and data pertaining to participants’ smoking status were obtained through the administration of a questionnaire. Respondents were provided with the options ‘Smoker’ and ‘Non-smoker’ to indicate their tobacco product usage status.

### Statistical analysis

Statistical analysis was performed using the SAS System software package (SAS Institute INC., North Carolina, USA). Taking into account the set level of statistical significance (0.05), the desired statistical power (0.80) and a moderate influence factor (effect size, d=0.5), the minimum sample size for the Wilcoxon Rank Sum test was 134 subjects divided into two groups, and for multiple regression analysis with, for example, 10 predictors, 118 respondents. The required sample size was calculated using G*Power software (Heinrich-Heine University Düsseldorf, Düsseldorf, Germany).

Descriptive statistics were employed to present data distribution through tabular and graphical representations. Numerical data were summarized using medians and interquartile ranges, while categorical data were presented with absolute and relative frequencies. To assess the normality of numerical variable distributions, the Shapiro-Wilk test was utilized. Relationships between numerical variables were evaluated using Spearman’s correlation coefficient. Differences in normally distributed numerical variables between independent groups were examined using Student’s t-test. In situations where deviations from a normal distribution were observed, the Wilcoxon Rank Sum test was applied.

In addition to the above tests, we also conducted adjusted multivariate logistic regression analysis. This analysis aimed to examine the influence of several factors on the probability of smoking. The model’s significance and explanatory power were evaluated using appropriate statistical tests and measures. All statistical tests were two-tailed.

## RESULTS

We enrolled a total of 164 subjects in the study, with 81 subjects (49.4%) categorized as smokers, while the remaining participants were non-smokers. Notably, the sample of smokers included a higher representation of women. Comprehensive demographic characteristics of the subjects in relation to smoking status are presented in Table 1.

**Table 1:** Demographic and physical characteristics of the subjects, Special Hospital for Medical Rehabilitation in Daruvarske Toplice, Croatia, 2019 (N=164)

*Characteristics*	*Smokers*		*Non-smokers*		*p*
	*n*	*Median (IQR)*	*n*	*Median (IQR)*	
**Age** (years)	81	53 (49–56)	83	54 (50–58)	0.138^†^
**Sex**					0.011^‡^
Women	17		6		
Men	64		77		
**Physical measurements**					
Weight (kg)	81	92 (78–103)	83	89 (80–98)	0.674^§^
Height (cm)	81	178 (172–182)	83	180 (174–184)	0.035^§^
Body mass index (kg/m^2^)	81	29 (26–32)	83	28 (26–29)	0.091^§^
Waist circumference (cm)	81	96 (89–107)	83	97 (90–102)	0.945^§^

IQR: interquartile range.

† Wilcoxon rank sum test.

§ t-test for independent samples.

‡ chi-squared test.

We conducted a detailed comparison of body composition parameters between smokers and non-smokers. Both groups exhibited similar average muscle tissue weight [t-test for independent samples: smokers, median=62 (IQR: 53–68); non-smokers, median=62 (IQR: 57–68) (p=0.125)] and comparable proportions of trunk adipose tissue in total adipose tissue [Wilcoxon Rank Sum test: smokers, median=51 (IQR: 51–51); non-smokers, median=51 (IQR: 51–51) (p=0.541)].

Smokers displayed a slightly higher mean percentage of fat tissue compared to non-smokers [t-test for independent samples: smokers, median=27% (IQR: 24–31); non-smokers, median=25% (IQR: 22–28) (p<0.001)]. This finding is substantiated by the data presented in Table 2.

**Table 2:** Comparison of the musculoskeletal system of smokers and non-smokers Special Hospital for Medical Rehabilitation in Daruvarske Toplice, Croatia, 2019 (N=164)

*Variable*	*Smokers*		*Non-smokers*		*p*
	*n*	*Median (IQR)*	*n*	*Median (IQR)*	
Muscle tissue (kg)	81	62 (53–68)	83	62 (57–68)	0.125^§^
Fat tissue (%)	81	27 (24–31)	83	25 (22–28)	<0.001^§^
Trunk adipose tissue proportion in total adipose tissue (%)	81	51 (51–51)	83	51 (51–51)	0.541^†^
The difference between the Gaia estimated age and the patient’s actual age	81	2 (0–3)	83	0 (-2–2)	0.003^†^

IQR: interquartile range.

† Wilcoxon rank sum test.

§ t-test for independent samples.

We observed a statistically significant difference in the Gaia estimated age and the actual age of the subjects, with smokers showing a higher discrepancy [Wilcoxon Rank Sum test: smokers, median=2 (IQR: 0–3); non-smokers, median=0 (IQR: -2–2) (p=0.003)]. Detailed results and statistical outcomes are elaborated upon in Table 2.

We conducted a multivariate logistic regression analysis to assess factors associated with current smoking status. The outcomes of this analysis are presented in Table 3. Our findings reveal that two independent variables related to current smoking status were: female gender (AOR=0.055; 95% CI: 0.004–0.072, p=0.027) and the difference between Gaia age estimate and actual age (AOR=1.631; 95% CI: 1.066–2.496, p=0.024). The model demonstrates overall statistical significance (χ^2^=24.27; df=3; p<0.001) and passed the Hosmer-Lemeshow test (p=0.68). The collective model explained between 13.8% (Cox and Snell) and 18.3% (Negelkerke) of the variability in current smoking status and accurately classified 64% of the cases.

**Table 3:** Prediction of smoking probability (adjusted multivariate logistic regression), Special Hospital for Medical Rehabilitation in Daruvarske Toplice, Croatia, 2019 (N=164)

*Variable*	*AOR*	*95% CI*	*p*
Sex	0.055	0.004–0.722	**0.027**
Fat tissue (%)	0.847	0.654–1.097	0.207
The difference between the Gaia estimated age and the patient’s actual age	1.631	1.066–2.496	**0.024**
Constant			0.139

AOR: adjusted odds ratio. Factor variable: sex. Covariates: fat tissue, and difference between the Gaia estimated age and the patient’s actual age. Statistical significance at p<0.05.

## DISCUSSION

Numerous studies have proven that smoking tobacco products has a harmful effect on the human body. In the beginning, the focus was on researching the cause-and-effect relationships between smoking and the diseases it causes, but lately the focus has been on the health that smoking takes away from us. The results of our research showed that smokers have a significantly higher percentage of adipose tissue compared to non-smokers, despite the fact that the mass (kg) of adipose tissue did not differ between the two groups. Chiolero et al.^19^ found that smokers have a paradoxically lower BMI, but their WHR is higher compared to non-smokers. WHR is considered a relevant measure of adipose tissue distribution, i.e. the amount of visceral fat, which is associated with the development of cardiovascular diseases, metabolic syndrome, and diabetes. For this reason, it is used as an anthropological measure in most studies that link the effects of smoking and the distribution of fat tissue in the body^10,20-22^. Taking the results of their literature review into account, the results of our research, which describe a significantly higher percentage of fat tissue in smokers compared to non-smokers, correlate positively with other research in the field. Clair et al.^23^ directly measured the percentage of fat tissue in the body by bio-impedance. The result of that research was that the percentage of adipose tissue increases proportionally with the amount of cigarettes smoked, but a higher percentage of adipose tissue in smokers compared to non-smokers was not described. Research by Lei et al.^24^ showed that already one month after smoking cessation, there is a regression of epigenetic changes, which are considered a relevant indicator of biological aging. The annual average worldwide spending on cosmetic products is around 500 billion dollars. This demonstrates the human obsession with the search for eternal youth. Serri et al.^25^ conducted a study in 2010 on the effect of smoking cessation on biological age, which was assessed by dermatological signs of aging, and the quantified results were astonishing: quitting smoking reduced biological age by as much as 13 years. Also, in our study, the difference between the GAIA 359 PLUS biological age estimate in relation to the actual age was significantly higher in smokers than in non-smokers. As the device works on the principle of measuring the impedance of tissues when alternating current is passed through them, it is clear that the assessment of biological age depends on body composition. It is important to emphasize that only healthy smokers participated in this research, which can be a direct indicator of how smoking alone causes changes in body composition. This would mean that timely cessation of smoking can directly affect the normalization of biological age in relation to chronological age.

### Limitations

While our study’s contributions are valuable, we must acknowledge its limitations. The cross-sectional design, while suitable for exploring associations, restricts our ability to establish causality. However, the use of the GAIA 359 PLUS device for estimating biological age adds a layer of objectivity to this marker. Nonetheless, it is important to note that even with advanced technology, there remain inherent limitations in accurately capturing the complex process of aging. Additionally, it is essential to consider that minor statistical differences in certain body composition parameters, while statistically significant, may not always translate into clinically significant differences, and this should be a point of consideration in the interpretation of our findings.

In terms of novelty, while the health risks associated with smoking are well-established, our study’s focus on its influence on body composition, including the estimation of biological age, adds a new perspective. By leveraging advanced technology, our data enhances the understanding of the intricate relationships between smoking, body composition, and aging.

In summary, our study’s limitations are balanced by the objectivity of the estimated biological age through the GAIA 359 PLUS device, and our findings contribute novel insights into the association between smoking, body composition, and aging. Further longitudinal investigations and broader participant cohorts have the potential to deepen these insights and expand our understanding.

## CONCLUSIONS

This research has shown that smokers, compared to non-smokers, have a significantly higher biological age than chronological age and a higher percentage of fat tissue. In contrast to numerous imperative reasons for smoking cessation, such as reducing the likelihood of developing chronic obstructive pulmonary disease as a multisystem disease that affects the whole body, not just the lungs, and the development of chronic metabolic diseases, the idea of ‘rejuvenation’, in the sense of reducing the biological age, could attract a greater number of smokers to make the decision to finally quit smoking with a non-invasive and attractive approach.

## Data Availability

The data supporting this research are available from the corresponding author on reasonable request.
